# Decoding RNA Editing Sites Through Transcriptome Analysis in Rice Under Alkaline Stress

**DOI:** 10.3389/fpls.2022.892729

**Published:** 2022-06-23

**Authors:** Obaid Rehman, Muhammad Uzair, Haoyu Chao, Muhammad Ramzan Khan, Ming Chen

**Affiliations:** ^1^Department of Bioinformatics, College of Life Sciences, Zhejiang University, Hangzhou, China; ^2^National Institute for Genomics and Advanced Biotechnology, National Agricultural Research Centre, Islamabad, Pakistan

**Keywords:** RNA editing, alkaline stress, rice, RNA-seq, *PPR*, *OZ1*, *MORF*

## Abstract

Ribonucleic acid editing (RE) is a post-transcriptional process that altered the genetics of RNA which provide the extra level of gene expression through insertion, deletions, and substitutions. In animals, it converts nucleotide residues C-U. Similarly in plants, the role of RNA editing sites (RES) in rice under alkaline stress is not fully studied. Rice is a staple food for most of the world population. Alkaline stress cause reduction in yield. Here, we explored the effect of alkaline stress on RES in the whole mRNA from rice chloroplast and mitochondria. Ribonucleic acid editing sites in both genomes (3336 RESs) including chloroplast (345 RESs) and mitochondria (2991 RESs) with average RES efficiency ∼55% were predicted. Our findings showed that majority of editing events found in non-synonymous codon changes and change trend in amino acids was hydrophobic. Four types of RNA editing A-G (A-I), C-T (C-U), G-A, and T-C were identified in treated and untreated samples. Overall, RNA editing efficiency was increased in the treated samples. Analysis of Gene Ontology revealed that mapped genes were engaged in many biological functions and molecular processes. We also checked the expression of pentatricopeptide repeat (*PPR*), organelle zinc-finger (*OZI*), and multiple organellar RNA editing factors/RNA editing factor interacting proteins genes in control and treatment, results revealed upregulation of *PPR* and *OZ1* genes in treated samples. This induction showed the role of these genes in RNA editing. The current findings report that RNA editing increased under alkaline stress which may contribute in adaptation for rice by changing amino acids in edited genes (88 genes). These findings will provide basis for identification of RES in other crops and also will be useful in alkaline tolerance development in rice.

## Introduction

Post-transcriptional mechanisms such as RNA editing, cause the change from DNA to RNA by means of insertion, deletions, and substitutions. This modification was first reported in mitochondrial genome many decades before (Benne et al., 1986), later on many studies identified the RNA editing sites in mitochondria and chloroplast (plastids) of animals and plants. In the case of plants, alteration in hereditary material through nucleotide conversion, deletion, or insertion leads to change in cytosine to uracil (C to U) in the messenger RNA of functional genes is the central dogma of RNA editing (Keller et al., 1999). Previously, RES was thought to be a proofreading mechanism because it corrected the mitochondrial mRNA through deletion/addition of uridine (Golden and Hajduk, 2006). With the passage of time and inventions of new sequencing techniques such as RNA sequencing (RNA-seq) enables the researcher to its importance in plants developments under the abiotic stress conditions (Duruvasula et al., 2019). This type of RNA editing is dominant, occurred in nucleus, cytosol, plastids, and mitochondria. The RNA editing plays a key role in the multiple plant growth and developmental processes, plant adaption to environmental influences, and signal transduction (Fujii and Small, 2011; Hammani and Giegé, 2014). Hence, RNA editing helps plant to cope with different environmental stresses, i.e., drought, heat, salt, etc. (Rieder et al., 2015; Rodrigues et al., 2017; Riemondy et al., 2018). In some cases, impaired RNA editing leads to abnormal plant ideotypes such as stunted plant growth and poor adoptability, even lethal phenotypes as impaired embryo development (Yan et al., 2018). This suggests that the RE plays a crucial role in growth and plant development. The RNA editing process has been already demonstrated in *Arabidopsis* (*Arabidopsis thaliana*), goatgrass (*Aegilops tauschii*), rice (*Oryza sativa*), maize (*Zea mays*), tobacco (*Nicotiana tabacum*), and grape (*Vitis vinifera*) (Wang et al., 2017; Chen et al., 2018; Rodrigues et al., 2020).

The most common editing types in non-flowering land plants are adenosine (A) to inosine (I) in tRNA, cytidine (C) to uridine (U) in messenger tRNA and RNA, and uridine (U) to cytidine (C) in mRNA (Chateigner-Boutin and Small, 2010; Zhang et al., 2016; Rasool et al., 2022). Protein function at the RNA level is maintain by RE events, e.g., failure of editing of the plastid ATPase alpha-subunit mRNA causes the pigment deficiency in tobacco cybrids (Sun F. et al., 2015). To find the RNA editing sites (RES), it is very important to get the knowledge of such post-transcriptional changes at the whole genome/transcriptome level of an organism to detect the mutations or polymorphism, and experimental evidence including high-throughput DNA/RNA sequencing is required. Previously, it is reported that RNA-seq can be used in the identification of RES in different crops (Chateigner-Boutin and Small, 2010; Eisenberg et al., 2010; Ichinose and Sugita, 2017).

The RNA editing in plants is primarily intervened by altering edifices including different altering factors, including organelle RNA recognition motif-containing *(ORRM)* protein, plant polyphenol oxidase *(PPO)*, *PPR*, *OZ*, and *RIP/MORF* (Takenaka et al., 2012; Wang et al., 2019). It is documented that an RNA editing involves the deamination of C-U with specified *PPR* and other genes both inside the nuclear genome (Yan et al., 2018). Studies showed the presence of motif of ∼35–40 amino acids in PPR proteins are the responsible for editing (Ichinose and Sugita, 2017). Many members of PPR family such as MEF9, CLB19, MEF32, and nad7-200 are required for the editing process (Takenaka et al., 2012). More and more members of PPR proteins are reported but the exact number of involved PPR is still missing (Schmitz-Linneweber and Small, 2008). Similarly, in kiwifruit, MORF2 and MORF9 are well documented; they interact with NADH4 and are very important for RNA editing of chloroplast genome (Xiong et al., 2022) while MORF8 interact with MORF1 and MORF2 (Zehrmann et al., 2015; Huang et al., 2019).

Rice is a very important cereal in the world, providing nutrition to the human. It faces different kinds of environmental stresses such as heat, drought, cold, salinity, insects, and pest attacks (Uzair et al., 2022). Being a high demand crop, it is necessary to increase the production of rice. Under salt stress, the processing of RNA due to RNA editing is still not well explored in rice. There are many types of RESs in chloroplast and mitochondrial transcriptomes but their effects in alkaline tolerant and susceptible genotypes is not reported. In this research, we analyzed the impact of alkaline stress on RNA processing by using RNA-seq data. We found more RNA editing events in the tolerant genotype, which might be due to stability of editing factors. Alkaline stress promotes the RNA editing at transcript level through the change in amino acids. These changes in amino acids in the genes will help the plants to cope with the environmental stresses.

## Materials and Methods

### Data Acquisition, Quality, and Mapping

Two rice genotypes Caidao (CD) which was alkaline-sensitive and WD20342 (WD) an alkaline-tolerant were assessed in control and alkaline environments (Li et al., 2018). The transcriptome-related data of these samples were publicly available and assessed from National Center Biotechnology Information (NCBI^1^) with the BioProject PRJNA414178.^2^ Reference genomes and annotation files of mitochondria (BA000029.3) and chloroplast (KT289404.1) were also accessed from NCBI in fasta format. Quality of sequence reads was assessed by using FastQC tool^3^ to identify low-quality reads and adapter sequences (Andrews, 2010). Hisat2 (v2.1.0) was used for index building and alignment of paired-end and clean reads with reference genome (Kim et al., 2019). Number of reads mapping to each gene were calculated with FeatureCounts (v2.0.0) (Liao et al., 2014). The fragments per kilobase of transcript per million mapped reads (FPKM) was calculated using the gene length and reads count mapped to that gene. Log fold change (LogFC) was applied on FPKM values and Heatmaps were generated by using “pheatmap” package of R.

### Differential Genes Expression Analysis

Two genotypes under control and stress were used to highlight differentially expressed genes and this was achieved by using the DESeq R package (Love et al., 2014); DESeq utilizes negative binomial distribution statistical model to predict differential expression. The *p*-values were adjusted (Benjamini and Hochberg, 1995). This was done to minimize the false discovery rate. Genes discovered by DESeq with a *p*-adjusted less than 0.05 were labeled as differentially expressed.

### Gene Ontology Analysis

Gene ontology (GO) analysis was performed to identify over-represented biological processes (BP), molecular functions (MF), and cellular components (CC) using the identified genes. The gene list was submitted to the ClueGO is a Cytoscape^4^ plug-in that combines Gene Ontology to produce a well-organized GO/pathway annotation (Shannon et al., 2003; Bindea et al., 2009). Gene ontology terms with *p*-values less than 0.05 were denoted significant enriched by DEGs.

### Analysis and Detection of RNA Editing Sites

RNA editing sites were predicted as previously described by Lv et al. (2018). In details, clean reads of RNA-seq data of the two rice (alkaline tolerant and susceptible) genotypes were aligned to the reference genomes of rice chloroplast and mitochondria. This was achieved with the help of SPRINT (SnP-free RNA editing Identification Toolkit) tool^5^ (Zhang et al., 2017). For SNP identification between RNA-seq and reference genomes of chloroplast and mitochondria, Genome Analysis Tool Kit (GATK^6^) which utilize BAM files were used for SNP calling (Van der Auwera and O’Connor, 2020). The overlapped findings of these two methods were used for further analysis. After this, samples of control of each genotype were used as a background, the SNPs difference between control and stressed samples were counted as RNA editing sites. The RNA-seq data from the control samples of the same genotype were used to avoid genotype-specific genomic SNP polymorphisms. At the end, the screening of data was done on the basis of (1) the mapped reads with more than five edited sites, (2) the ratio of edited reads/total mapped reads was more than 50%, and (3) RES were predicted in all three replicates. The Ensemble Variant Effect Predictor (VEP) tool^7^ with default parameters (chromosome number, start and end position, allele, and strand) was used to annotate the RNA editing site (McLaren et al., 2016). For this purpose, *O. sativa* specie was used.

### Expression Profiling of Selected Genes Through Real-Time PCR for Quantification

Seeds of rice genotype (IRRI6) were sterilized with 2.5% sodium hypochlorite for 35 min followed by three washes and placed in 37°C Owen for 1 day. Uniformly germinated seeds were grown under normal conditions (16-h light/8-h dark at 26 ± 2°C) for 2 weeks. For this purpose, 96-wells plates supported by a container filled with nutrients media (Yoshida) were used. After this, one batch was kept as a control and in other batch 0.5% Na_2_CO_3_ solution (pH = 11.3) was applied for 36 h to create alkaline stress. The leaf samples were collected in triplicates. The total RNA was extracted with the help of TRIzol method and complementary DNA was prepared by using reverse transcriptase-III, first strand cDNA Synthesis Kit (K1691, Thermo Scientific Revert Aid). Real-time PCR for quantification (qRT-PCR) of selected genes of *PPR*, *OZ1*, and *MORF/RIP* gene family members was performed by using StepOne RT-PCR (Applied Biosystems^®^ 7900 HT Fast RT-PCR). Three biological replicates from control and treated samples were used. *OsActin* was used as an internal control and for expression calculations 2^ΔΔ^ CT method was used (Uzair et al., 2021). List of gene-specific primers are provided in Supplementary Table 1.

## Results

### Alignment of RNA-Seq Data

**FIGURE 1:**
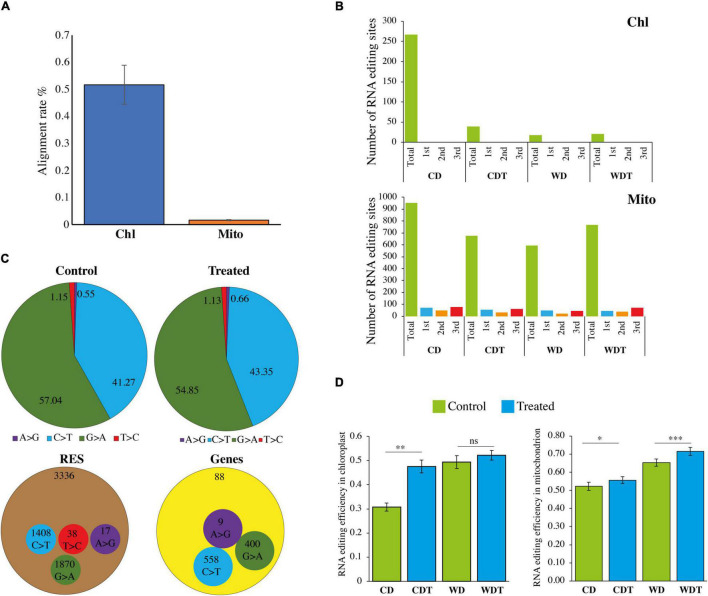
Summary of RNA-seq data. **(A)** Mapping rate of RNA-seq data of chloroplast (Chl) and mitochondria (Mito). **(B)** Codon position of RNA editing sites in two genotypes (CD, Caidao and WD, WD20342) of rice, chloroplast (Chl) genome, and mitochondrion (Mito) genomes. **(C)** Comparison of single nucleotide conversion in control and treated samples by RNA-seq approach. Values are shown by percentage. Number of total RES and edited genes. **(D)** Editing efficiency of all the RNA sites in rice chloroplast and mitochondrion genomes. For comparison, *t*-test was used. ^***^p < 0.001; ^**^p < 0.01; *p < 0.05 was used for significant while ns, non-significant.

Plants of two rice genotypes Caidao (CD) and WD20342 (WD) were raised under normal and stress conditions. The genome size of rice mitochondrial and chloroplast genomes is 490,520 and 134,556 bp with number of genes 81 and 129, respectively (Notsu et al., 2002; Asaf et al., 2017; Feng et al., 2021). The genome of mitochondria has a smaller number of genes as compared to chloroplast genome. An average of ∼2,915 reads were aligned with the reference mitochondrial genomes with percentage of 0.01% (0.002 std). Similarly, for chloroplast, the average ∼92,881 reads aligned with the mapping on reference 0.51% (0.07 std). In general, chloroplast have more genes as compared with mitochondria. We expected that mapping reads should more in chloroplast and we found the expected results (Table 1 and Figure 1A).

**TABLE 1:** Summary of mapping rate to mitochondria and chloroplast genomes.

					Mitochondria		Chloroplast	
Sample ID	Type	Total reads	Raw base (bp)	GC%	Uniquely mapped	Multiple mapped	Uniquely mapped	Multiple mapped
SRR6168973	CD1	17,553,050	2,632,957,500	54	3372 (0.02%)	7827 (0.04%)	109763 (0.63%)	11837 (0.07%)
SRR6168974	CD2	19,525,592	2,928,838,800	53	4008 (0.02%)	6170 (0.03%)	104658 (0.54%)	8880 (0.05%)
SRR6168975	CD3	16,981,675	2,547,251,250	55	5422 (0.03%)	15708 (0.09%)	185979 (1.10%)	29909 (0.18%)
SRR6168976	CDT1	13,856,046	2,078,406,900	52	2190 (0.02%)	5502 (0.04%)	65959 (0.48%)	10551 (0.08%)
SRR6168977	CDT2	13,536,683	2,030,502,450	52	3871 (0.03%)	3936 (0.03%)	106303 (0.79%)	7809 (0.06%)
SRR6168978	CDT3	18,341,629	2,751,244,350	53	2640 (0.01%)	6317 (0.03%)	98310 (0.54%)	10989 (0.06%)
SRR6168979	WD1	18,719,664	2,807,949,600	56	4546 (0.02%)	5188 (0.03%)	104072 (0.56%)	7306 (0.04%)
SRR6168980	WD2	20,898,805	3,134,820,750	57	1597 (0.01%)	2219 (0.01%)	49513 (0.24%)	5517 (0.03%)
SRR6168981	WD3	20,627,635	3,094,145,250	55	1120 (0.01%)	2209 (0.01%)	47692 (0.23%)	3782 (0.02%)
SRR6168982	WDT1	20,989,565	3,148,434,750	56	2561 (0.01%)	5109 (0.02%)	96742 (0.46%)	11753 (0.06%)
SRR6168983	WDT2	24,277,815	3,641,672,250	55	2309 (0.01%)	5561 (0.02%)	90751 (0.37%)	14109 (0.06%)
SRR6168984	WDT3	20,905,666	3,135,849,900	55	1431 (0.01%)	2898 (0.01%)	54833 (0.26%)	8597 (0.04%)

*Three replicates of two genotypes of rice (CD, Caidao and WD, WD20342) under control and alkaline treatment.*

### Identification and Characterization of RNA Editing Sites

To build the sequencing profundity and unwavering quality of editing sites, the subsequent bam documents of three replicates of each genotype under each condition were converged for resulting distinguishing proof of RESs. After the SNPs calling, we found total 3,336 RNA editing sites in both genomes including 345 in chloroplast and 2,991 in mitochondria, respectively (Table 2). We notice that some of the genes expressed in specific conditions such as control and alkaline stress. The chloroplast genome showed only seven edited genes. The data revealed that the higher editing sites were G-to-A (150 out of 345); 137 out of 345 editing sites were C-to-T, the second most common type in chloroplast. Similarly, T-C were 38 and A-G were 20 out of 345 (Supplementary Table 2). In chloroplast genome, in control samples (CD = 3 genes and WD = 1 gene) showed a larger number of edited genes while in stress conditions (CDT = 2 genes and WDT = 1 gene), less genes were edited (Table 3). In mitochondrial genome, two types of editing sites C-to-T and G-to-A were found; most edited sites 1,720 out of 2,991 editing sites were G-to-A and the second most common type of editing, 1,271 out of 2,991, was C-to-T (Supplementary Table 3). Mitochondrial genome showed that total 81 edited genes. In control samples (CD = 19 genes and WD = 19 genes) showed a smaller number of edited genes, while in stress conditions (CDT = 23 genes and WDT = 20 genes) more genes were edited in mitochondrial genome (Table 3). These results showed that more genes were edited in mitochondrial genome as compared to chloroplast and similarly under stress condition as compared to control samples. This increase is might be due to biasness of the sequencing; mapping rate increase in stress samples as compared to control one which might lead to new editing sites. Second, upregulation of genes (*PPR* and *OZ1*) involved in RNA editing.

**TABLE 2:** Summary of identified RNA editing sites in chloroplast and mitochondrion genome of rice under alkaline stress.

Organelle	Samples	Total
Chloroplast	CD	267
	CDT	39
	WD	18
	WDT	21
Mitochondrial	CD	953
	CDT	677
	WD	594
	WDT	767

*CD, Caidao in control; CDT, Caidao in treatment; WD, WD20342 in control; WDT, treated.*

**TABLE 3:** Mitochondrial RNA editing sites in genes, type of editing, and their occurrence in four samples of rice.

	Sample	Gene	Type	Occurrence	Sample	Gene	Type	Occurrence
Chloroplast	CD	psbC	C/T	03	CDT	rpoC2	A/G	04
		psbC	A/G	05		ndhB	G/A	07
		ndhB-2	C/T	36				
	WD	ndhB-2	C/T	05	WDT	EPlORYSAT000373811	C/T	03
Mitochondria	CD	nad3	C/T	21	CDT	rps3	C/T	09
		rps12	C/T	08		pseudo-rpl16	C/T	03
		rsp2	G/A	07		nad3	C/T	14
		nad4	C/T	09		rps12	C/T	10
		cox2	C/T	21		rps2	G/A	10
		atp6	G/A	20		nad4	C/T	07
		nad5	G/A	13		cox2	C/T	20
		rps4	G/A	05		apt6	G/A	22
		rps19	C/T	08		nad5	G/A	06
		nad4L	C/T	11		nad1	G/A	17
		cob	G/A	05		rps4	G/A	10
		nad1	G/A	10		atp9	C/T	05
		mat-r	G/A	12		rps19	C/T	04
		rps1	G/A	08		nad4L	C/T	04
		ccmFn	G/A	22		cob	G/A	06
		ccmFc	C/T	21		mat-r	G/A	06
		nad9	C/T	06		rps1	G/A	05
		nad2	C/T	09		ccmFn	G/A	06
			G/A	06		ccmFc	C/T	16
		ccmB	C/T	28		apt1	C/T	05
						nad9	C/T	06
						nad2	C/T	12
						ccmB	C/T	11
	WD	rps3	C/T	10	WDT	rps3	C/T	15
		nad3	C/T	11		pseudo-rpl16	C/T	10
		rsp12	C/T	04		nad3	C/T	15
		nad4	C/T	06		rps12	C/T	05
		cox2	C/T	21		rps2	G/A	13
		atp6	G/A	12		nad4	C/T	12
		nad5	G/A	12		cox2	C/T	19
		rps4	G/A	06		atp6	G/A	18
		atp9	C/T	04		nad5	G/A	15
		rps19	C/T	03		nad1	G/A	18
		nad4L	C/T	11		rps13	G/A	07
		cob	G/A	10		rps4	G/A	11
		nad1	G/A	09		cob	G/A	11
		mat-r	G/A	06		mat-r	G/A	07
		ccmFn	G/A	12		ccmFn	G/A	16
		ccmFc	C/T	15		ccmFc	C/T	11
		atp1	C/T	04		cox1	C/T	09
		nad9	C/T	06		nad9	C/T	13
		nad2	C/T	10		nad2	C/T	19
			G/A	02			G/A	12
						ccmB	C/T	10

### Annotation of Editing Sites

We employed VEP tool for the identification of characteristics summary of RNA editing sites. The results revealed that there was no RNA editing site detected in the genes of chloroplast while in mitochondrial genome RNA editing in third codon position was mainly occurred (Figure 1B) whereas editing in first codon position was second the most common type. In synonymous variant, we did not find any codon changes at the second position of the amino acid. We compared the control samples with treated ones and found that both have four (A > G, C > T, G > A, and T > C) types of editing (Figure 1C), respectively. In control samples, we found 57.04% G > A, and 41.27% C > T types of editing. Similarly, in the treated samples, G > A was 54.85% and C > T was 43.35%. We found a total of 3,336 RNA editing sites in both genomes and has four types of RNA editing types (Figure 1C). The G > A was the most dominant type 1,870 out of 3,336 RESs. Similarly, C > T was the second most type 1 408 out of 3 336 RES, followed by T > C (38 RES), and A > G (17 RES). The results showed that there were 88 total edited genes and these genes have three types C > T (558 RES), G > A (400 RES), and A > G (9 RES) of editing types (Figure 1C). In addition, larger editing events resulted in as degenerative codon. We found that most of the neutral amino acid changes to hydrophobics followed by neutral to hydrophilic (Supplementary Table 4). Previously, Yan et al. (2018) and Zhang et al. (2020) also described that RNA editing increased the hydrophobicity of the newly synthesized proteins.

Based on the genetic properties, we divided RNA editing sites in the mitochondrial genome into six different types (Supplementary Table 5). These types include upstream variants, downstream variants, missense variants, synonymous variants, start lost, and stop gained. The results revealed that CD owned the 44% downstream gene variants followed by upstream gene variant 27%, missense 17%, synonymous variants 11%, start lost 1%, and stop gained 2%. In CDT, 50% downstream gene variants followed by upstream gene variant 25%, missense 14%, synonymous variants 10%, and 4% stop gained. Similarly, in tolerant genotype WD have 51% downstream gene variants, 26% upstream gene variant, 14% missense variants, 9% synonymous variants, start lost 1%, and 2% stop gained under control condition. Similarly, under stress condition, WDT have 46% downstream gene variants, 36% upstream gene variant, 10% missense variants, 9% synonymous variants, and 2% stop gained. Interestingly, we did not find start lost in CDT and WDT.

### Ribonucleic Acid Editing Efficiency With and Without Stress

We checked the statistics of RNA editing efficiency. In total, the average editing efficiency of RESs was about 0.54. In chloroplast, the genome efficiency was 0.31, 0.48, 0.49, and 0.52 (Figure 1D). While in mitochondria, RE efficiency was 0.52, 0.56, 0.65, and 0.72 (Figure 1D). In chloroplast, there was a significant (*p* < 0.01) difference in CD untreated and treated samples while no difference was found in WD treated and untreated samples. In mitochondria, both genotypes CD and WD under treatment showed induction in editing efficiency. Overall, the tolerant genotype showed increased efficiency.

### Cluster Analysis of RNA Editing Efficiency

**FIGURE 2:**
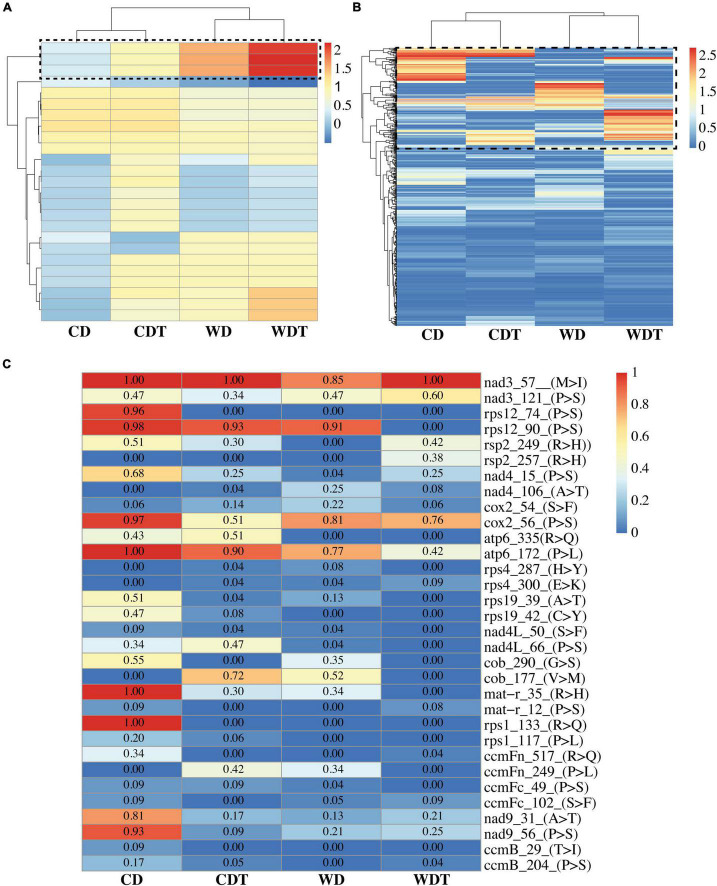
Heatmap of RNA editing efficiency of all the RNA sites in two genotypes of rice (CD, Caidao and WD, WD20342), **(A)** Chloroplast genome; **(B)** mitochondrion genome. The *x*-axis shows the genotypes under control (CD and WD) and treated (CDT and WDT) while the *y*-axis shows the editing sites. Black dotted box representing the RNA editing sites increased efficiency. **(C)** Reduced RNA editing efficiency in genes of mitochondria. Values insides the boxes were showing RNA editing efficiency. The *x*-axis shows the genotypes under control (CD and WD) and treatment (CDT and WDT). Gene names with amino acid position and type of changes were shown on the *y*-axis.

We performed cluster profiling of the chloroplast (Figure 2A) and mitochondria (Figure 2B) genomes in samples of CD, CDT, WD, and WDT. The results revealed that RE efficiency was increased in the CDT and WDT under treatment. This increased in RE efficiency is related to tolerance of the genotypes and also showed that RE is a process which helps the plants to survived in the stress environment. These results are the further confirmation of the previous results (Figure 1D). All the information related to RE efficiency was presented in Supplementary Table 6.

### Effects of RNA Editing Efficiency on Genes

Findings of annotations showed that 81 genes out of total 88 genes were annotated. All the annotated genes were belonging to mitochondrial genome (Figure 2C and Supplementary Tables 5, 7). The genes in chloroplast were not showed any annotation. In mitochondrial genome, many genes were showing more editing sites, especially ccmB showed 49 editing sites in all the samples of two genotypes. All the editing types were C-T (C-U) showing decreasing trend from CD to WDT; amino acid changes were A (Ala)–T (Thr), P (Pro)–L (Leu), P (Pro)–S (Ser), and T (Thr)–I (Ile). Overall trend for these amino acids was neutral to hydrophobic. Similarly, the ndh2 showed two types of RNA editing: (1) C-T (C-U = 50) and (2) G-A (20) but not annotated. Different genes such as NADH dehydrogenase gene family (nad3/4/4L/9), ribosomal genes family (rps1/2/3/4/12/19), ATPase family (atp1/6), heme trafficking system membrane genes family (ccmB/ccmFn/ccmFc), mitochondrial cytochrome *c* oxidase gene family (cox1 and 2), and maturase R (matR) were detecting in this study and heatmap of randomly selected genes were generated (Figure 2C).

It was previously reported that the ribosomal genes family played a role in protein synthesis, growth development in plants, and proper functioning of chloroplasts (Zhou et al., 2015; Uzair et al., 2021). Members of the mitochondrial cytochrome *c* oxidase (COX) helps in aerobic energy development (Timón-Gómez et al., 2018). The NADH dehydrogenase genes involved in plant growth in response against the environmental stress (Hashida et al., 2009). Similarly, in chloroplast genome, ndhB-2 gene was edited which help in fixation of C-to-U back mutations through the DNA proofreading mechanism (Martín and Sabater, 2010). In tobacco *ndhB* genes improves the photosynthetic ability, stress resistance, and energy transformation (Horváth et al., 2000). The molecular function of maturase R (*matR*) is not fully clear but it converts the edited amino acid to its original form. In angiosperm, one *matR* was reported in the fourth intron of *nad1* gene (Sultan et al., 2016). Subsequently, alteration in amino acids sites due to evolution or RES might adjusts through maturase implementation.

### Identification of A-I and C-U RNA Editing Sites

We checked the RESs in chloroplast and mitochondria of four samples (Supplementary Tables 2, 3). Both genotypes (CD and WDT) were used under control and treatment which making four samples (CD, CDT, WD, and WDT). In the chloroplast genomes, the genotypes CD showed total 10 A-I RES all were A-G associated with psbC gene and 122 RES were C-T (C-U). In CDT samples, total identified A-I RES were 10, from this 10 RES were A-G associated with the gene rpoC2 and 11 RES were C-T (C-U). A total of 9 out of 20 RES were A-I in both of these genes. We did not find any RES in WD samples under control and under treatment. We found only five C-T (C-U) RES in WD and 16 C-T (C-U) RES in WDT. We also checked the mitochondrial genome also, but unluckily we cannot find any A-I RES in any sample. However, we found 386 C-T (C-U) RES in CD, 311 RES in CDT, 243 RES in WT, and 331 RES in WDT.

### Gene Ontology Analysis of Mapped Genes

**FIGURE 3:**
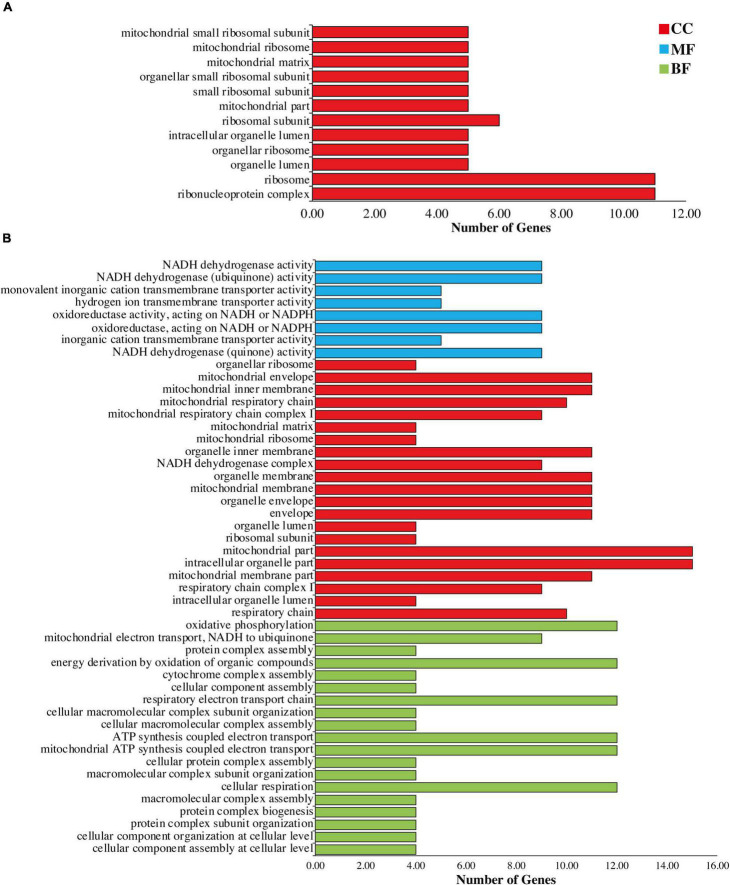
Gene Ontology annotation of genes of **(A)** chloroplast and **(B)** mitochondria. Red color showing cellular components (CC), blue color representing molecular functions (MF), and green color showing biological functions (BF).

To check the functions of the genes in which RESs were detected, all REGs in all sample were annotated to the terms in the GO database. Results revealed that 12 and 48 significantly enriched functions identified in chloroplast and mitochondria, respectively. In chloroplast we found only 12 cellular components (Figure 3A). Most enriched components in each sample including organelle ribosome and mitochondrial small ribosomal subunit were associated with genes of ribosomes such as *rpl16*, *rpl2*, *rpl20*, *rps11*, *rps12*, *rps14*, *rps16*, *rps19*, *rps2*, *rps3*, and *rps7*.

In mitochondria genome, most enriched processes were mitochondrial ribosome, NADH dehydrogenase activity, cytochrome complex assembly, protein complex assembly, oxidative phosphorylation, cellular component organization at cellular level, ribosomal subunit, mitochondrial membrane, ATP synthesis coupled electron transport, and mitochondrial electron transport NADH to ubiquinone (Figure 3B and Supplementary Table 8).

### Expression Profiling of *PPR*, *OZ1*, *MORF/RIP*, and RNA Edited Genes

**FIGURE 4:**
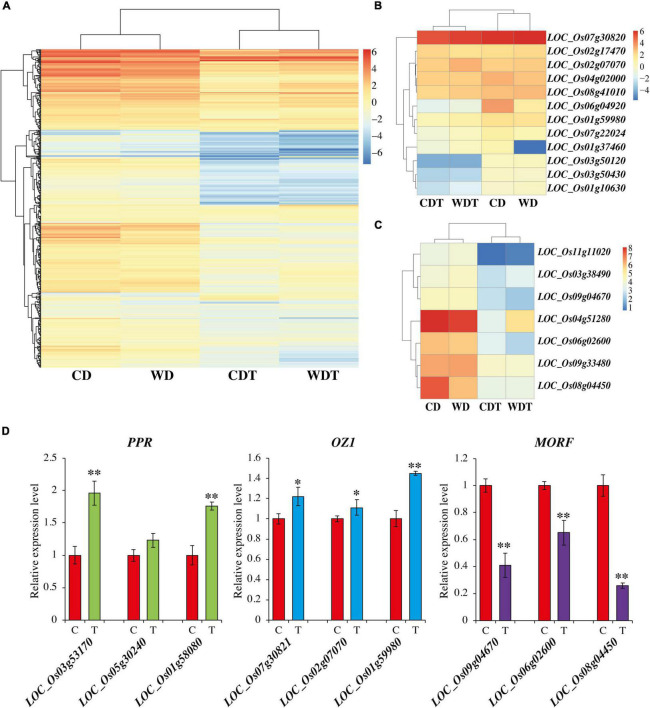
Expression of *PPR* genes **(A)**, *OZ1* genes **(B)**, and *MORF/RIP* genes **(C)** in two genotypes of rice (CD, Caidao and WD, WD20342) under control and treatment. **(D)** Expression analysis of *PPR*, *OZ1*, and *MORF/RIP* genes through qRT-PCR in control (C) and treatment (T). Mean of three replicates with ±SE were used. For comparison *t*-test was used (^**^p < 0.01 and *p < 0.05).

For the comparison among the edited genes and genes involved in editing was done through transcriptome analysis. We checked the expression of RNA edited genes, but there were no significant differences observed under treated and untreated samples (Supplementary Figure 1). This indicates that alkaline stress only influences the RE events and has no role in the expression of these genes. To find the reason of reduction of RE efficiency in edited genes we analyzed the expression of genes involved in RE-like pentatricopeptide repeat proteins (*PPR*), *Organelle Zinc finger 1* (*OZ1*), and multiple organellar RNA editing factors (*MORF/RIP*) genes. After the blast results, we found a total of 482 genes of *PPR* family, 12 genes of *OZ1* family, and 7 *MORF/RIP* genes in rice. Previously, it was reported that rice have 477 *PPR* genes but one more study reported 491 *PPR* genes in rice (O’Toole et al., 2008; Chen et al., 2018). Similarly, seven *MORF* genes are reported in rice (Zhang et al., 2019). The results showed that there was a reduction of expression with the treatment (Figure 4A). Some of the *PPR* genes were also upregulated (Supplementary Table 9). The expression of *OZ1* genes and *MORF/RIP* genes were also reduced for most of the genes (Figures 4B,C). To confirmed these findings, we selected already reported PPR genes *LOC_Os03g53170*, *LOC_Os05g30240*, and *LOC_Os01g58080* (Chen et al., 2018), and *MORF* genes *LOC_Os09g04670*, *LOC_Os06g02600*, and *LOC_Os08g04450* (Zhang et al., 2019). While OZ1 genes *LOC_Os07g30821*, *LOC_Os02g07070*, and *LOC_Os01g59980* were selected randomly for expression analysis through qPCR. The results showed that there was induction in *PPR* and *OZ1* genes under alkaline stress, while the genes of *MORF* were downregulated (Figure 4D). These results were with the agreement of previous studies. Overall, results showed that there was a positive association among the RNA editing and PPR genes.

## Discussion

The RNA editing (RE) is a post-transcriptional mechanism in which transcripts modified by different mutations types such as insertion, deletion, and substitutions. The RE was firstly reported in *kinetoplastid protozoa* and then identified in higher plants and animals (Maslov et al., 1994). It can change the gene structure which cause the RNA splicing (Farré et al., 2012). For proper functioning of the RNA, its structure is very important. The thermodynamics of secondary structure are well studied under salt stress (Serra and Turner, 1995; Zuker, 2003) and folding of RNA is very sensitive to salt concentrations (Tan and Chen, 2011). Editing events played role in RNA degradation and also helps in microRNA regulation. The tertiary structure of the RNA helps in the arrangement of the secondary structure (Thirumalai, 1998). Our study suggests that the RNA editing due to secondary and tertiary structures are affected by alkaline stress. For confirmation of molecular mechanism of this RNA editing needs future experimentations. The RNA editing improves the plant growth and adoptability (Liu et al., 2013; Sun T. et al., 2015). Previously, it is reported that changes in mitochondrial genome at specific sites cause harmful effects such as growth, seed production, development, and fertility (Toda et al., 2012; Yap et al., 2015). Some studies also reported that abiotic and biotic stresses also affect RE and it have role in evolution of the species (Nakajima and Mulligan, 2001; Fujii and Small, 2011; Hammani and Giegé, 2014). In grapes it was proved that RNA editing is sensitive to temperature (Zhang et al., 2020). Similarly, in wheat RNA editing improve the drought tolerance (Rasool et al., 2022). Furthermore, in maize RNA editing is necessary for seed germination and development (Liu et al., 2013).

With the innovation of new sequencing approaches such as RNA sequencing, RESs were distinguished mainly in living species, particularly in plants. Current study, reported many RESs, and the statics of altering types demonstrated that RE normally happens as G-A, T-C, C-U, and A-I changes in deciphered areas of cellular organs mRNAs. We found that changes in coding regions of the proteins were found at third and first positions which changed the physiochemical properties of the amino acids (Khosravi and Jantsch, 2021; Din et al., 2022). Most of the amino acids were changed to hydrophobic which are agreement to the previous research (Zhang et al., 2020). This indicates that for the production of functional proteins RNA editing acts as a proofreading mechanism in plants (Ichinose and Sugita, 2017). In this study, induction of RE in alkaline tolerant genotypes proved the relationship of abiotic stress and RNA editing. Previously many studies showed the relationship of abiotic stress and RNA editing (Ruwe et al., 2013; Hajrah et al., 2017). It is reasonable that RNA editing helps the plants to survive in the stressed environment by regulating gene functions. This increase is necessary for keeping the homeostasis among the functions of the genes and RNA editing is proved to be very important for crop vigor (He et al., 2018). Similar findings also reported in soybean, in which RNA editing efficiency increased due to salt stress (Rodrigues et al., 2017). In other study of NADH dehydrogenase subunit 7 (nad7) in barley, salinity enhanced the RNA editing efficiency (Ramadan et al., 2021).

In plants, the RNA editing plays a crucial role for transcript maturation by conversion and insertion/deletion. Different genes families such as pentatricopeptide repeat proteins (*PPR*), organelle zinc finger 1 (*OZ1*), organelle RNA recognition motif-containing protein (*ORRM*), protoporphyrinogen IX oxidase (*PPO*), and multiple organellar RNA editing factors/RNA editing factor interacting protein (*MORF/RIP*) genes are reported in the RNA editing (Zhang et al., 2014; Haag et al., 2017; Tian et al., 2019; Wang et al., 2019). Out of these gene families, *PPR*, *OZ1*, and *MORF/RIP* are the important part of editosome and they are directly or indirectly involved in RNA editing (Okudaira et al., 2021). Pentatricopeptide repeat proteins played important role in editing and more than 450 members are reported in the different crops such as Arabidopsis and rice (Lurin et al., 2004; Chen et al., 2018). Pentatricopeptide repeat proteins are reported that they played role in growth, development, and found in plastids and mitochondria. The mutants of *PPR* showed different abnormal defects related to growth (Saha et al., 2007). The target sites of the RNA are predefined and they have specific sites for the attachment of *PPR*. So, the RNA editing is the best tool to understand this mechanism (Manna, 2015). In our study, the expression of PPR genes was increased under stress condition which indicates that RES are responsive to alkaline stress. It was previously reported that *MORF* genes family have role in abiotic stress and involved in RNA editing (Wang et al., 2019). Pentatricopeptide repeat proteins contains a C-terminal domain and interact with *MORF* genes (Takenaka et al., 2012; Brehme et al., 2014). Latest research reported that *MORF* involved in plant growth, development, stress response such as survival of seedling rice, drought resistance in poplar, and pathogen resistance in tobacco (Wang et al., 2019; Zhang et al., 2019; Yang et al., 2020). Our results showed the downregulation of *MORF* genes under alkaline stress and agreement with the findings of Zhang et al. (2019) and Xiong et al. (2022). Similarly, *NbMORF8* which is present in the mitochondria and negatively enhance the immunity to *Phytophthora* pathogens in tobacco (Yang et al., 2020). More research needs to be involved to clarify the role of such gene family’s involvement or not in RNA editing.

## Conclusion

The post-transcriptional changes such as C-U are conserved among animals as well as in plants. We used alkaline treated and control samples for comparison. We found four types of RNA editing A-G (A-I), C-T (C-U), G-A, and T-C, and most editing events were identified in non-synonymous codons which increased the hydrophobicity. Overall, RNA editing efficiency was increased due to stability of editing factors in the alkaline stressed samples. Furthermore, RNA editing changes the amino acids in the edited genes which help the plants to cope abiotic situations. These outcomes will contribute to improve understanding the molecular mechanism of dynamics of gene regulation at post-transcriptional level in rice. These findings will be useful in designing alkaline tolerance breeding programs in rice.

## Data Availability Statement

The RNA-seq data was publically available at the followimg link https://www.ncbi.nlm.nih.gov//bioproject/PRJNA414178.

## Author Contributions

OR, MU, and HC performed the analysis. OR and MU wrote the original draft. MK provided research facilities and guided during the whole study. MC supervised the whole study, reviewed, and edited the manuscript. All authors contributed to the article and approved the submitted version.

## Conflict of Interest

The authors declare that the research was conducted in the absence of any commercial or financial relationships that could be construed as a potential conflict of interest.

## Publisher’s Note

All claims expressed in this article are solely those of the authors and do not necessarily represent those of their affiliated organizations, or those of the publisher, the editors and the reviewers. Any product that may be evaluated in this article, or claim that may be made by its manufacturer, is not guaranteed or endorsed by the publisher.

## Footnotes

1
https://www.ncbi.nlm.nih.gov

2
https://www.ncbi.nlm.nih.gov//bioproject/PRJNA414178

3
https://www.bioinformatics.babraham.ac.uk/projects/fastqc/

4
https://cytoscape.org/

5
http://sprint.tianlab.cn

6
https://gatk.broadinstitute.org/hc/en-us

7
https://asia.ensembl.org/info/docs/tools/vep/index.html
