# Supplementary material for: Decoding RNA Editing Sites Through Transcriptome Analysis in Rice Under Alkaline Stress
Source: Front Plant Sci. 2022 Jun 23;13:892729. doi: 10.3389/fpls.2022.892729 (PMC9260663; doi:10.3389/fpls.2022.892729)
Supplement: Supplementary file 1 [file Data_Sheet_1.ZIP › Supplementary Files/Supplementary Figure S1.docx]

Supplementary Material

Decoding RNA Editing Sites through Transcriptome Analysis in Rice under Alkaline Stress

Obaid Ur Rehman^1,2^, Muhammad Uzair^2^, Haoyu Chao^1^, Muhammad Ramzan Khan^2^, Ming Chen^1, *^

^1^Department of Bioinformatics, College of Life Sciences, Zhejiang University, Hangzhou, China

^2^National Institute for Genomics and Advanced Biotechnology, Park Road Islamabad, Pakistan.

*** Correspondence:**Ming Chen
[mchen@zju.edu.cn](mailto:mchen@zju.edu.cn)


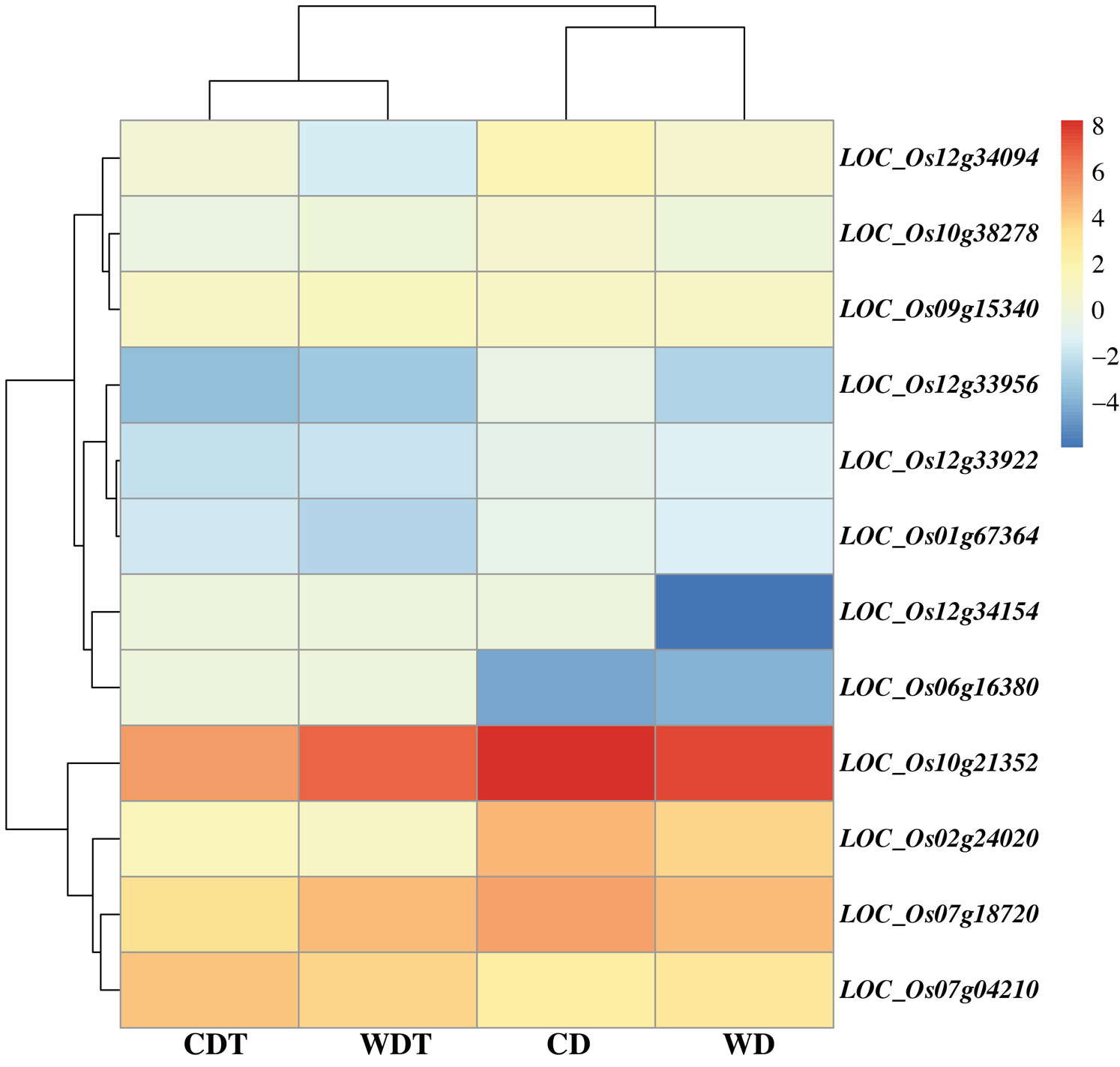


**Supplementary Figure S1.** Heatmap of selected mapped genes.
